# Sonication of revised hip and knee prostheses detects occult infections, improves clinical outcomes and prevents re - revisions. A case series study

**DOI:** 10.1016/j.infpip.2022.100232

**Published:** 2022-07-12

**Authors:** Argyris C. Hadjimichael, Athanasios F. Foukas, Angelos Kaspiris, Dimitris Vasileiou, Spyros Kamariotis, Antonios Stylianakis, Elias S. Vasiliadis, Olga D. Savvidou, Athanasios Antonopoulos

**Affiliations:** a Department of Orthopaedics, St Mary's Hospital, Imperial College Healthcare NHS Trust, Praed Street, W2 1NY, London, UK; b Third Department of Orthopaedic Surgery, “KAT” General Hospital of Athens, Nikis 2, 14561, Kifissia, Greece; c Laboratory of Molecular Pharmacology, Department of Pharmacy, School of Health Sciences, University of Patras, Patras 26504, Greece; d Orthopaedic Department, Mediterranean Hospital of Cyprus, Stygos 9, 3117, Limassol, Cyprus; e Microbiology Department, “KAT” General Hospital of Athens, Nikis 2, 14561, Kifissia, Greece; f Third Department of Orthopaedic Surgery, National and Kapodistrian University of Athens, Faculty of Medicine, “KAT” General Hospital of Athens, Nikis 2, 14561, Kifissia, Greece; g First Department of Orthopaedic Surgery, National and Kapodistrian University of Athens, Faculty of Medicine, Attikon University Hospital, Athens,1 Rimini Street, Chaidari,12462, Athens, Greece

**Keywords:** Arthroplasty, Hip, Knee, Periprosthetic infection, Sonication, Oxford score

## Abstract

**Introduction:**

Periprosthetic joint infection (PJI) is a devastating complication occurring in 1–2% of primary and up to 10% of revised total hip and knee arthroplasties (THA and TKA) impairing patient's quality of life. Occult infections are underdiagnosed, sub-treated and sub-clinically experienced by patients. This study aimed to correlate patients' clinical outcomes with early antibiotic treatment based on use or non-use of a sonication technique on explanted prostheses.

**Methods:**

33 patients with revised THA or TKA were retrospectively evaluated. Clinical outcomes were assessed via Oxford hip or knee scores, and correlated with administration or not of antibiotic treatment based on sonication results.

**Results:**

According to laboratory findings the patients were divided in the following three groups: 1. Septic loosening (conventional cultures and/or sonication positive), 2. Aseptic loosening (conventional cultures and sonication negative) and 3. Occult loosening (conventional cultures negative, sonication not performed). The average Oxford score was poor (27.9/60) for the septic, excellent (43.8/60) for the aseptic and intermediate (37.7/60) for the occult group. Additionally, conventional cultures were negative, but sonication-positive, in 6 individuals with patient-related risk factors (male gender, BMI > 30 kg/m^2^, diabetes, hypertension, steroids and rheumatoid arthritis).

**Conclusions:**

Sonication represents a valuable diagnostic technique to guide administration of effective antibiotic treatment for patients, especially for detection of persistent post-revision occult infections. We recommend the systematic investigation of revised prostheses with a sonication technique, but especially in cases with risk factors for infection who it is suspected may have occult loosening.

## Introduction

Total hip and total knee arthroplasties (THA, TKA) resolve painful symptoms and functional restrictions in daily activities of patients suffering from osteoarthritis [1]. It is anticipated that demand for primary joint replacement will rapidly increase, up to 284% for THA and up to 401% for TKA, in the United States up to 2040 [1].

Periprosthetic joint infection (PJI) is a devastating complication that occurs in 2–3% of primary joint arthroplasties leading to poor quality of life and necessity for a revision surgery [2]. In most of cases, patients who are diagnosed with a periprosthetic hip or knee infection undergo either one-stage or two-stage revision surgery without impact on the risk for recurrent infections [3,4]. Unfortunately, revision surgeries due to septic failure are correlated with five-fold higher mortality rate compared with aseptic revisions [5]. Compared to primary joint replacements, revision arthroplasties have potentially a 7-fold higher risk of being complicated by a new PJI leading to further increase in morbidity and mortality [6].

*Staphylococcus* species and low-virulence pathogens colonize implant surfaces and develop complex 3D-communities which produce highly hydrated and self-produced extracellular matrix formatting a glycocalyx biofilm [7]. Mature biofilms make detection and treatment of microorganisms more difficult and they become up to 1000 times more resistant to antimicrobial agents [7]. Therefore, bacteria can detach from biofilms, activate the host's immune system and cause implant loosening [8]. Early diagnosis of occult infections remains crucial for the sufficient treatment of PJIs. Unsuspected or occult PJIs remain a diagnostic pitfall causing subtle symptoms in patients and contributing to chronic “aseptic” loosening of prostheses [8]. In 2018 the European Bone and Joint Infection Society introduced diagnostic criteria for PJIs [9]. However, PJIs can be misdiagnosed for several reasons such as false negative conventional cultures [10], preoperative antibiotic use, wrong culture media, inappropriate incubation time and microbial death during transportation to the microbiology laboratory [11].

The sonication technique uses application of long-wave ultrasounds which radiate through the liquid medium of explanted implants [12]. This detaches bacteria from biofilms and increases the number of culturable bacterial cells. Latent and low virulent bacteria might be recognized only by sonication technique but not detected with conventional culturing [12]. Recent literature has proposed that the combination of sonication technique with conventional fluid/tissue cultures exhibits additional diagnostic value in clinical practice [13,14].

The aim of our study was to evaluate the contribution of sonication-guided antibiotic treatment in the quality of life in patients with revised infected arthroplasties. Consequently, to investigate whether sonication technique could early detect subclinical occult infections and prevent from chronic loosening and future re-revision surgeries.

## Materials and methods

### Study characteristics

This is a single-centre retrospective case series study which involved the medical records from 33 patients with one revision surgery of infected TKA or THA between February 2012–June 2019. During this period, 74 patients had a revision surgery for their primary hip or knee arthroplasty due to mechanical failure, impingement, periprosthetic fracture and repetitive dislocations of hip or knee replacements. In 33 out of 74 cases the main reason for implant failure was considered a potential septic loosening, therefore further microbiological tests were performed (conventional cultures/sonication) to evaluate the presence or absence of infectious bacteria. Consequently, 33 patients were included in the present retrospective study. Patients were divided in two groups, those with positive periprosthetic cultures (n=15) and those with negative periprosthetic cultures (n=18) after retrieval of hip and knee prostheses. In addition, we retrospectively interpreted the presence of the following patient-related risk factors prior to revision surgery: demographic data such as gender (male), age, BMI, long-term prescription of corticosteroids and comorbidities such as diabetes, hypertension and rheumatoid arthritis. Thirteen out of 18 cases with negative periprosthetic cultures had such patient-related risk factors. The sonication technique was not performed in 6 out of these 13 patients due to intraoperative findings, such as purulence, inflammation of periprosthetic tissues and implant macroscopic appearances indicative of a septic joint.

All patients were interviewed by two independent orthopaedic surgeons using the Oxford hip or knee questionnaires if at least two years have passed from their last surgery. Periprosthetic tissue/fluid cultures and sonication fluid cultures were assessed by two independent microbiologists. Neither orthopaedic surgeons who interviewed patients were informed about patient's laboratory results nor microbiologists were aware of their clinical outcomes. All data were collected from a third independent orthopaedic surgeon. A written consent was obtained from all patients and the study is in accordance with the Helsinki Declaration of 1964. The present study was approved by the Institutional Review Board of our hospital (protocol code: 5914, date of approval: 07/05/2018).

### Inclusion and exclusion criteria

Inclusion criteria were the available medical reports from sonicated fluid cultures of explanted prostheses and con-ventional periprosthetic tissue cultures between February 2012–June 2019.

Exclusion criteria were lack of sufficient tissue culture samples (at least two) and retrieval of hardware other than prostheses or prosthetic components. We also excluded patients whose tissues or components were obviously contaminated after removal, or who had received antimicrobial agents during the 14 days preceding revision surgery. Finally, we excluded revised THA and TKA from non-osteoarthritic patients.

### Diagnostic criteria for periprosthetic infections (confirmed, likely and unlikely)

Diagnosis of periprosthetic joint infection was established after review of clinical and laboratory findings by orthopedic surgeons and microbiologists, based on the criteria of European Bone and Joint Infection Society (EBJIS) [9]. An infection was *confirmed* if at least one of the following criteria was present: (1) sinus tract communicating with the joint, or direct visualization of prosthesis; (2) leucocyte count > 3,000/μL; (3) PMN >80%; (4) positive alpha-defensin; (5) ≥2 positive intraoperative tissue/fluid samples containing the same microorganism; (6) sonication positive for >50 CFU/ml of any organism; (7) ≥5 neutrophils in ≥5 high-power fields (HPF) (400x magnification); (8) microorganisms seen on direct microscopy [9].

Infection was *likely* to exist if ≥2 of the following criteria were met: (1) radiological findings of loosening five years after implantation; (2) wound healing problems; (3) history of fever or bacteraemia; (4) purulence around prothesis; (5) CRP>10mg/l; (6) leucocyte count > 1,500/μL; (7) PMN >65%; (8) single tissue/fluid sample culture-positive; (9) sonication positive for >1 CFU/ml of any organism; (10) ≥5 neutrophils in a single HPF; (11) positive WBC scintigraphy [9].

### Diagnostic criteria for aseptic loosening

Aseptic loosening (infection unlikely to exist) was defined according to the following criteria: (1) implant disfunction attributed to reasons other than infection (e.g., fracture, breakage, malposition); (2) leucocyte count ≤ 1.500 cells/μl; (3) PMN ≤ 65%; (4) tissue/fluid cultures negative; (5) sonication cultures negative; (6); No neutrophils/HPF; (7) negative three-phase isotope bone scan [9].

### Surgical technique

Revision surgeries of THA (posterolateral approach) and TKA (medial parapatellar approach) were performed by two experienced consultant surgeons using the hip direct lateral (Hardinge) approach [15,16]. When EBJIS criteria for *confirmed* or *likely* periprosthetic infections were found the septic bone and infected soft tissues were totally removed. After radical debridement, all infected tissues and joint fluids were sent for culture. Explanted protheses were went to the laboratory as described below.

When EBJIS criteria were *unlikely* to support the diagnosis of infection, soft tissue and joint fluid were sent for culture without sonication of explanted prostheses. However, if patient-related risk factors for infection were reported (male gender, BMI > 30 kg/m^2^, diabetes, hypertension, steroid therapy, rheumatoid arthritis) sonication was performed [17].

### Periprosthetic cultures

Two to six (median n=4) periprosthetic tissue samples with inflammatory characteristics from bone-cement/bone-prosth-esis interfaces were collected. Tissue specimens were individually homogenized in 3mL trypticase for 1 min using a mortar and pestle. 0.1 mL aliquots of the homogenates were inoculated onto aerobic (SBA) and anaerobic sheep blood agar (ASBA) plates and 1mL was inoculated into thioglycolate broth. Cultures were incubated at 35 ± 1°C for 10 days. A terminal subculture was performed from the thioglycolate broth onto SBA and ASBA, and incubated at 35 ± 1°C for a further 5 days. Each unique microbial colony was identified, and antimicrobial susceptibility testing performed, using standard methods. Cultures were considered positive if the same microorganism was present in ≥ 2 periprosthetic tissue samples [18,19].

### Sonication fluid cultures

**Figure 1:**
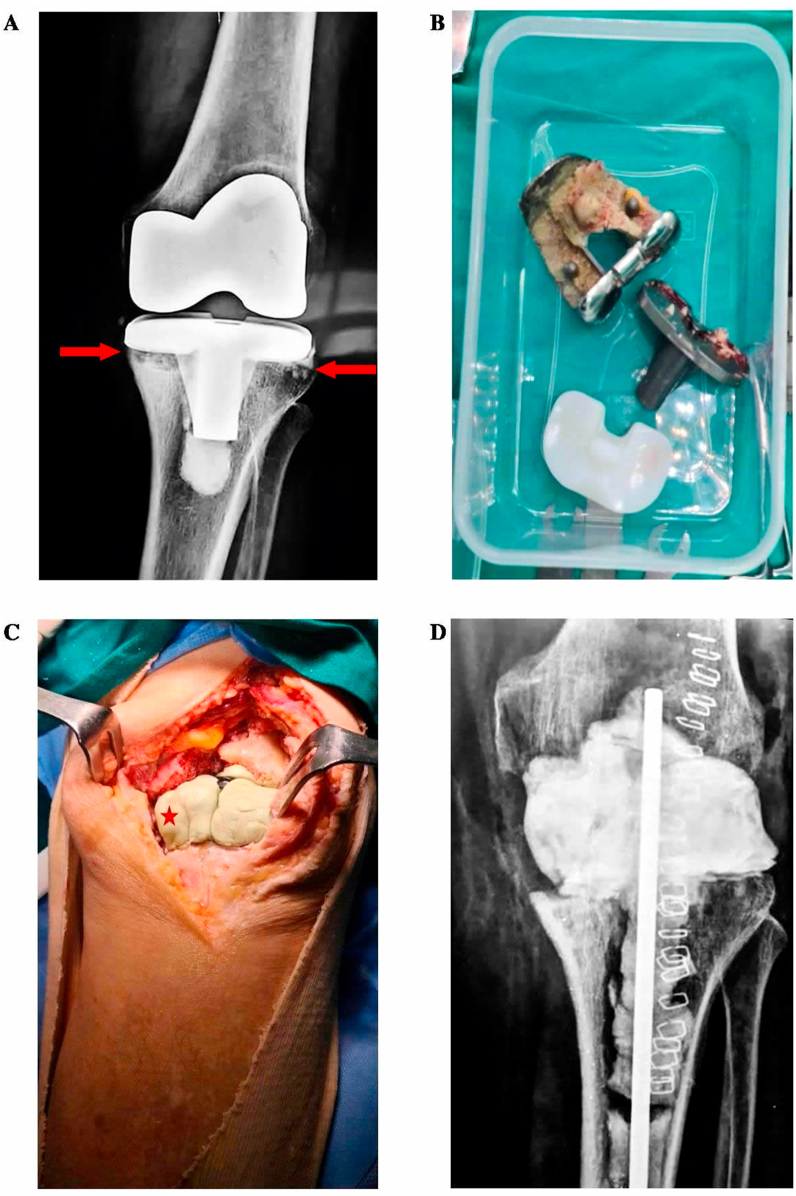
**Collection of explanted prostheses in a two-stage TKA. (A)** X-ray of a patient with loosening if his TKA. The arrows show the radiolucent appearance of loosening around the tibial prosthesis. **(B)** Explanted prostheses were transferred to the microbiology de? lkjbpartment for sonication in a sterile solid air-tight container. **(C)** The femoral and tibial tunnels were filled with vancomycin-impregnated cement beads until second stage of revision. The asterisk shows the cement filling the gap **(D)** The final x-ray after the first phase of revision. The patient received antibiotic therapy according to conventional and sonication fluid cultures. After a 2-year follow up his Oxford Score was found 29/60.

Prosthetic components were aseptically explanted and transferred to the microbiology laboratory in sterile solid air-tight containers (Lock & Lock; Vertrag AG, Stafa, Switzerland) (Figure 1) [20]. Sterile Ringer solution (volume ranging from 50-200mL depending on implant size) was added to the container in a laminar airflow biosafety cabinet, covering 85–90% of the volume of larger implants, or the entire volume of smaller ones. The container with the implant was vortexed for 30 s, followed by sonication for 1 min (frequency: 40 kHz, power density: 0.22 W/cm^2^), a BactoSonic ultrasound bath (Bandelin GmbH, Berlin, Germany), as determined by a calibrated hydrophone. The container was then vortexed for a further 30 s to remove residual microorganisms and to homogeneously distribute them in the sonication fluid. 0.1 mL or 1.0 mL aliquots of sonicate fluid were inoculated onto solid media into broth as for periprosthetic cultures. As well as identify, and performing antibiotic susceptibility tests, colonies were counted to determine the number of CFU/mL of sonication fluid.

### Clinical evaluation

We used the Oxford Hip Score (OHS) and the Oxford Knee Score (OKS) in our study. Both scores are validated patient-reported outcome measures (PROMs) to evaluate THA and TKA outcomes [[21], [22], [23]]. Patients were interviewed by two independent orthopaedic surgeons for scoring.

### Statistical analysis

Statistical analysis was performed using MedCalc statistical software v. 20.0.1 (MedCalc Software Ltd. Ostend, Belgium). The Mann-Whitney U test was used to compare OHS and OKS between patients with septic, aseptic and occult implant loosening. One-way analysis of variance was used to compare the Oxford Hip and Knee scores between patients with positive and negative sonication fluid cultures and periprosthetic tissue cultures. Results were considered significant when *P* value was < 0.05. Positive and negative predictive values regarding sonication fluid cultures and periprosthetic tissue cultures for each detected microorganism were also calculated.

## Results

The study enrolled 33 patients (63.6% female, n = 21 and 36.4% male, n = 12); median age at time of revision arthroplasty 73 years (6 patients were aged ≤ 60 and 27 patients were 61–80 years). 20 (60.6%) patients had a revised THA and 13 (39.4%) a revised TKA.

### Laboratory results

#### Periprosthetic tissue and fluid cultures

Bacteria were isolated from 15/33 (45.4%) of patients, with 18 (54.5%) therefore being defined as having “aseptic loosening”.

#### Sonication fluid cultures

**Figure 2:**
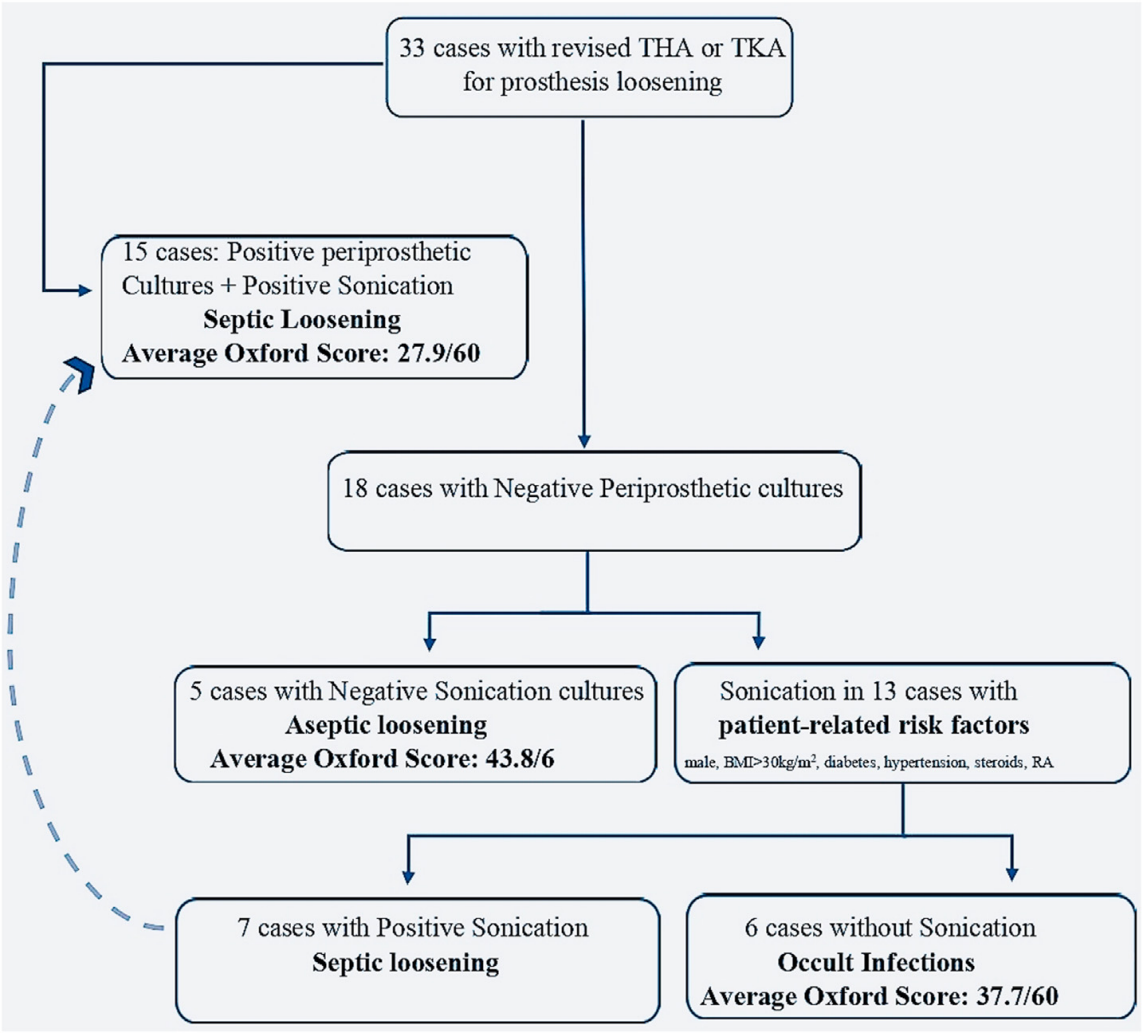
**Algorithm for inspected cases.** Laboratory findings vs clinical outcomes. The flow chart depicts the algorithm we followed in our retrospective cohort study and the number of evaluated cases in each group. Implant loosening was assessed in laboratory via conventional and sonication fluid cultures and they were correlated with clinical outcomes with OHS and OKS.

Sonication was performed for all 15 patients with positive periprosthetic tissue/fluid cultures, and for 13/18 patients who had negative conventional cultures, but clinical risk factors for infection (Figure 2). Seven (53.8%) of these 13 patients in the “aseptic” loosening group had microbial growth in the sonication fluid cultures, leading their infection statues to be reclassified as “occult infection”. Amongst the total of 22 cases with septic loosening, seven different microorganisms were isolated from periprosthetic tissue cultures. There were 7 instances of inconsistency between the two culture methods (Table I). The positive and negative predictive values of sonication fluid cultures and periprosthetic tissue cultures for each microorganism are shown in Table II.

**Table I:** Clinical and laboratory results from each patient

Case NO.	Type of loosening	Oxford SCORE/60	Sonication fluid cultures	Periprosthetic tissue/fluid culture
1	**Aseptic**	41	Negative	Negative
2	**Aseptic**	48	Negative	Negative
3	**Aseptic**	42	Negative	Negative
4	**Aseptic**	47	Negative	Negative
5	**Aseptic**	41	Negative	Negative
6	**Occult**	40	Not performed	Negative
7	**Occult**	40	Not performed	Negative
8	**Occult**	36	Not performed	Negative
9	**Occult**	37	Not performed	Negative
10	**Occult**	36	Not performed	Negative
11	**Occult**	37	Not performed	Negative
12	**Septic**	27	*Staphylococcus epidermidis*	*Staphylococcus epidermidis*
13	**Septic**	28	1.*Staphylococcus aureus* (MRSA) 2.*Staphylococcus epidermidis*	*Staphylococcus epidermidis*
14	**Septic**	29	*Staphylococcus epidermidis*	1.*Staphylococcus epidermidis* 2.*Streptococcus agalactiae*
15	**Septic**	25	*Staphylococcus aureus* (MRSA)	*Staphylococcus aureus* (MRSA)
16	**Septic**	22	*Staphylococcus epidermidis*	*Staphylococcus epidermidis*
17	**Septic**	27	*Staphylococcus lugdunensis*	*Staphylococcus lugdunensis*
18	**Septic**	25	1.*Staphylococcus aureus* 2.*Staphylococcus warneri* 3.*Staphylococcus lugdunensis*	1.*Staphylococcus aureus* 2.*Staphylococcus warneri*
19	**Septic**	24	*Staphylococcus epidermidis*	*Staphylococcus epidermidis*
20	**Septic**	23	1.*Staphylococcus aureus* 2.*Staphylococcus epidermidis*	1.*Staphylococcus aureus* 2.*Staphylococcus epidermidis*
21	**Septic**	21	*Staphylococcus aureus*	1.*Staphylococcus aureus* 2.*Streptococcus agalactiae* 3.*Staphylococcus epidermidis*
22	**Septic**	18	*Staphylococcus warneri*	*Staphylococcus warneri*
23	**Septic**	21	1.*Staphylococcus aureus* 2.*Staphylococcus epidermidis* 3.*Staphylococcus hominis*	*Staphylococcus hominis*
24	**Septic**	27	*Staphylococcus aureus*	*Staphylococcus aureus*
25	**Septic**	26	1.*Staphylococcus aureus (MRSA)* 2.*Streptococcus agalactiae*	1.*Staphylococcus aureus* (MRSA) 2.*Streptococcus agalactiae*
26	**Septic**	27	*Staphylococcus aureus* (MRSA)	*Staphylococcus aureus* (MRSA)
27	**Septic**	38	*Staphylococcus epidermidis*	Negative
28	**Septic**	28	1.*Staphylococcus epidermidis* 2.*Staphylococcus lugdunensis* 3.*Acinetobacter baumannii*	Negative
29	**Septic**	37	*Staphylococcus epidermidis*	Negative
30	**Septic**	39	*Streptococcus agalactiae*	Negative
31	**Septic**	36	1.*Staphylococcus epidermidis* 2.*Staphylococcus hominis*	Negative
32	**Septic**	37	1.*Staphylococcus epidermidis* 2.*Staphylococcus aureus* (MRSA)	Negative
33	**Septic**	29	1.*Staphylococcus epidermidis* 2.*Staphylococcus hominis*	Negative

**Table II:** Positive and Negative predictive values of sonication fluid cultures and periprosthetic tissue cultures for the detection of each microorganism that were isolated in our study. PPV: Positive Predictive Value, NPV: Negative Predictive Value

Microorganism	Sonication fluid cultures		Periprosthetic tissue cultures	
	PPV (%)	NPV (%)	PPV (%)	NPV (%)
*Staphylococcus epidermidis*	100	87,5	100	50
*Staphylococcus aureus*	100	100	100	71,4
*Staphylococcus aureus (MRSA)*	100	100	100	55,6
*Staphylococcus lugdunensis*	100	100	100	42,9
*Staphylococcus warneri*	100	100	100	100
*Streptococcus agalactiae*	100	50	100	66,7
*Staphylococcus hominis*	100	100	100	42,9

### Clinical outcome results

**Figure 3:**
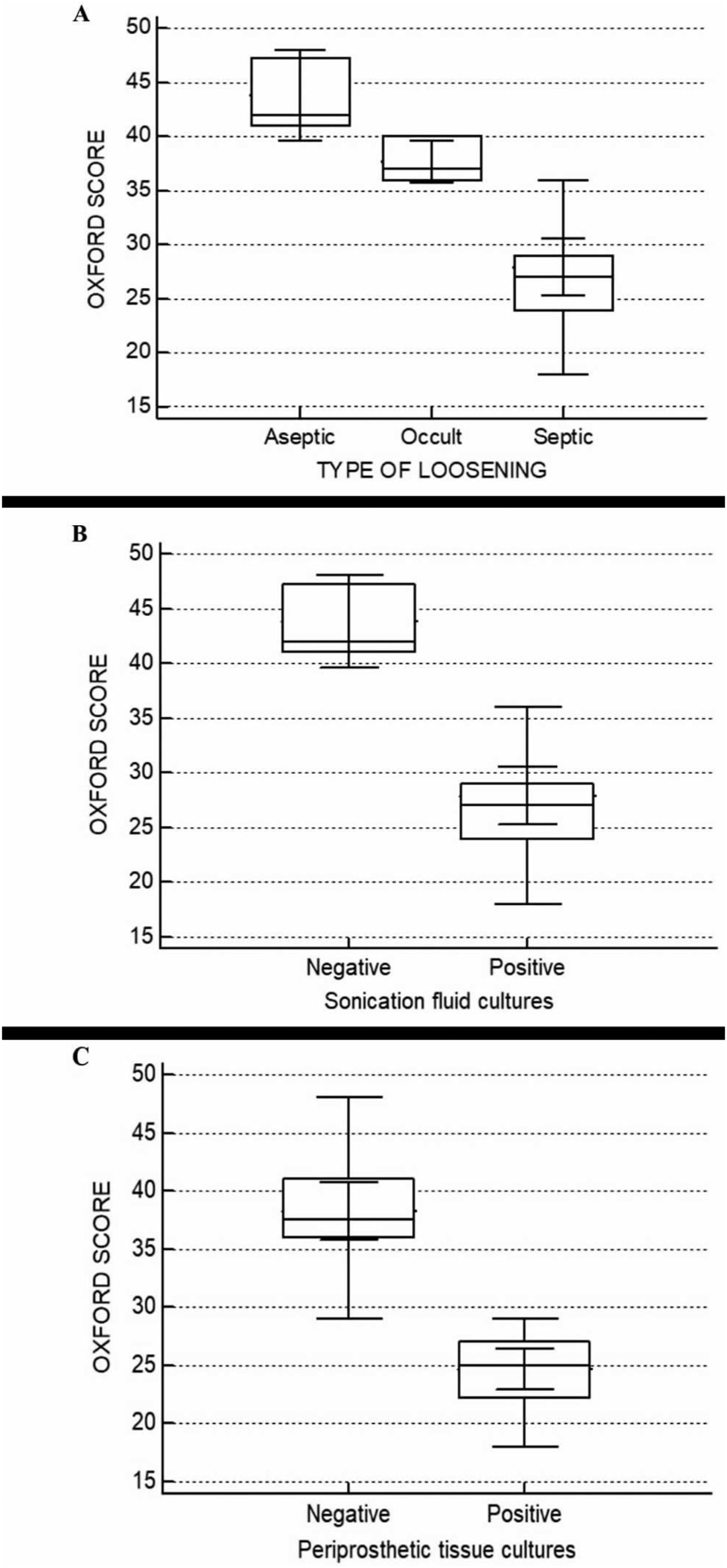
**Multiple comparison graph of Oxford scores between: (A) three types of loosening, (B) patients with negative and positive sonication fluid cultures (B), (C) patients with negative and positive periprosthetic tissue cultures. (A)** Oxford score was measured higher in patients with aseptic loosening, intermediate in patients with occult loosening and lower in patients with septic loosening. These differences were statistically significant (*P*<0,001). **(B)** Oxford score was measured higher in patients with negative sonication fluid cultures and lower in patients with positive sonication fluid cultures. This difference was statistically significant (*P*<0,001). **(C)** Oxford score was found higher in patients with negative periprosthetic tissue cultures and lower in patients with positive periprosthetic tissue cultures. This difference was statistically significant (*P*<0,001).

Mann-Whitney U test revealed a significant difference (*P*<0.0001) between patients with septic, occult and aseptic implant loosening. Patients with aseptic loosening reported the highest Oxford scores, while patients with septic loosening reported the lowest Oxford score (Figure 3A).

Likewise, one-way analysis of variance showed a statistically significant difference (*P*<0,0001) between patients with negative sonication fluid cultures or negative periprosthetic tissue cultures and those with positive cultures. Patients with negative cultures were measured with higher Oxford scores (Figures 3B and 3C). Overall, 22/33 (66.6%) patients had positive implant cultures, and were characterized as “septic”. These patients reported the worst average Oxford score of 27.9/60, indicating their poor quality of life. The five patients with negative tissue and sonication fluid cultures (characterized as “aseptic”) had the best average Oxford score of 43.8/60. Interestingly, the 6 patients with negative tissue cultures, and sonication not been performed, had an intermediate average Oxford Score of 37.7/60. The correlation between laboratory results and Oxford scores is presented in Table I.

## Discussion

The present study assesses the impact of sonication-guided antibiotic therapy on clinical outcomes and quality of life in patients with revised THA or TKA by using PROMs post-operatively. To our knowledge, this is the first study which correlates sonication with OHS and OKS after removal of infected prostheses.

THA and TKA are the ''gold standard'' for the treatment of advanced osteoarthritis. Demand for these is expected to increase due to a growing burden of osteoarthritis-related risk factors such as obesity, sports injuries and ageing [24]. Unfortunately, joint replacements can lead to complications that deteriorate patients' quality of life, and PJIs in particular are associated with serious disability and even mortality [25]. Currently, the usual and widely accepted conventional method of diagnosis is culture of periprosthetic tissue and fluid [26]. However, presence of biofilms, and the effect of pre-operative antibiotic exposure can render standard cultures falsely-negative PJIs [26]. Resulting undertreatment might increase the morbidity and impact on patients' quality of life, which in turn places economic burdens on hospitals and health systems in general [25,26].

OHS and OKS are useful predictors of early revision following primary THA and TKA in line with re-revision surgeries [27]. According to Rothwell *et al.*, every one-unit decrease in Oxford scores as early as six-months after primary joint replacement can increase the risk for revision to 9.7% for THA and 9.9% for TKA [27]. Likewise, Kalairajah *et al.*, demonstrated that a poor score of <27/60 is associated with an increased risk for revision (THA: 7.6%,TKA: 7%) compared with an excellent score of >34/60 (THA:0.7%, TKA:0.7%) within two years [28].

We found that patients with aseptic loosening reveal the best Oxford Clinical Scores and those with septic loosening the worst. Interestingly, patients with risk factors for infection and negative conventional cultures, but positive sonication fluid cultures, had poor Oxford scores <27.9/60, indicating that periprosthetic tissue cultures alone will miss a number of PJIs. Notably, 6 cases with patient-related risk factors for infection had negative conventional cultures but sonication fluid cultures were not performed. These patients had intermediate Oxford scores (37.7/60) between septic (27.9/60) and aseptic loosening cases. We suggest that some of these patients may have had missed low-grade occult infections that would have been detected with sonication, informing targeted antibiotic therapy.

Beyond doubt, the patient's satisfaction reduces after a revision surgery affecting their clinical outcomes and PROMs [26]. Patients experience long-term poorer quality of life and their implants might require re-revision. For instance, patients with a primary THA and TKA report better daily satisfaction compared to those with revision surgery [26]. Interestingly, the literature is conflicting on whether patients with revised arthroplasties due to septic loosening experience inferior clinical and functional outcomes compared with those who had revised implants for aseptic loosening [29,30]. In our study, patients of the septic loosening group had significant worse Oxford Scores and functional outcomes compared with those without infected implants.

### Limitations of our study

Our study has important limitations. It is a retrospective single-centre study with a small sample size, meaning that the findings may not be generalizable, and they are subject to possible bias [31]. Inclusion of patients with both one or two-stage hip revision could have affected the outcomes reported in Oxford scores as every new surgical intervention may cause additional psychological burden on patients. Finally, we interviewed patients at least 2 years from the last surgery but not at a fixed postoperative time, which may have introduced recall bias.

## Conclusions

Sonication fluid cultures represent an accurate, easy, cheap and sensitive diagnostic modality that is complementary to periprosthetic tissue/fluid cultures. The application administration of sonication-guided antibiotic treatment might benefit patients otherwise in low-risk for infection due to EBJISs criteria. In accordance with our results, the extended use of sonication in individuals with patient-related risk factors such as male gender, BMI > 30 kg/m^2^, diabetes, hypertension, steroid therapy and rheumatoid arthritis is remarkable and leads to early detection of occult infections and preventions of re-revisions.

### Credit author statement

Argyris C. Hadjimichael - methodology, formal analysis, writing-original draft, Athanasios F. Foukas - methodology, formal analysis, supervision, project administration, Angelos kaspiris - conceptualization, methodology, data curation, writing-review & editing, Dimitrios Vasileiou - methodology, investigation, Spyros Kamariotis - investigation, Antonios Stylianakis - investigation, supervision, project administration, Elias Vasiliadis - formal analysis, Olga D. Savvidou - conceptualization, supervision, Athanasios Antonopoulos - supervision, project administration.

## Institutional Review Board statement

The study was conducted according to the guidelines of the Declaration of Helsinki, and approved by the Institutional Review Board of “KAT” General Hospital of Athens (protocol code: 5914 and date of approval: 07/05/2018).

## Informed consent statement

Informed consent was obtained from all subjects involved in the study.

## Conflict of interest statement

The authors have no conflicts of interest to declare.

## Funding

This research did not receive any specific grant from funding agencies in the public, commercial, or not-for-profit sectors.
